# Supplementary material for: Cyclin B/CDK1 and Cyclin A/CDK2 phosphorylate DENR to promote mitotic protein translation and faithful cell division
Source: Nat Commun. 2022 Feb 3;13:668. doi: 10.1038/s41467-022-28265-0 (PMC8813921; doi:10.1038/s41467-022-28265-0)
Supplement: Supplementary file 1 — Supplementary Information [file 41467_2022_28265_MOESM1_ESM.pdf]

Supplementary Information

Cyclin B/CDK1 and Cyclin A/CDK2 phosphorylate DENR to promote mitotic protein translation and faithful cell division

Clemm von Hohenberg et al.

Contents:

1) Supplementary Discussion p.2

2) Supplementary Figures...p. 5

3) Supplementary References ....p. 19

1) Supplementary Discussion

Testing of post-translational modifications on DENR•MCTS1 function using a translational reporter

In this and the following section we provide the reader for future reference the data we obtained on the other residues of the DENR•MCTS1 complex besides DENR Ser73.

We aimed to systematically test the role of all post-translational modifications on DENR•MCTS1 activity. To this end, we mutated all amino acid residues in DENR or MCTS1 that are known from public databases (e.g. phosphosite.org) to be the targets of phosphorylation, acetylation or ubiquitylation. We then tested whether these mutations affect DENR•MCTS1 function by expressing the mutants in DENR knockout or MCTS1-depleted cells. As a readout for DENR•MCTS1 function we assayed translation of a luciferase reporter containing a short upstream Open Reading Frame with a strong Kozak sequence (stuORF) (Suppl. Figure 8a), which we previously showed to be translated in a DENR•MCTS1-dependent manner [1, 2]. The only mutation that produced a strong effect on stuORF translation was the MCTS1[T117A,S118A] allele (Suppl. Figure 8b-c), which we previously reported to affect DENR•MCTS1 activity in Drosophila (corresponding to residues T118 and S119 in flies [1]). When we tested protein stability of these mutants using a timecourse of cycloheximide (CHX) which blocks protein synthesis, we noticed that MCTS1 Thr117 affects MCTS1 stability, with both the T117A single mutant and the T117A/S118A double mutant versions of MCTS1 being less stable than wildtype MCTS1 (Suppl. Figure 8d,e). This likely explains the reduced activity of these mutants. MCTS1[S118A] however, is even more stable than wildtype MCTS1 (Suppl. Figure 8e), yet has impaired activity (Suppl. Figure 8b), suggesting that phosphorylation of this site might be of interest for future

investigation. Worth mentioning is that we performed these experiments in HeLa cells in standard culture conditions, where some of these residues may not be modified by phosphorylated, acetylation, etc. In that case, mutation of the corresponding residue does not give a phenotype compared to the wildtype protein because the post-translational modification is also absent in the wildtype protein. Hence, in the future it may be worth testing this panel of mutants and our custom-made phosphoantibodies, also in other cell lines or environmental conditions. Furthermore, we used for this stuORF assay (Suppl. Figure 8b-c) asynchronous cells, explaining why the DENR S73A mutant, which is relevant only in mitosis (see main text), did not have a strong phenotype. Our data on all potential regulatory sites within the DENR•MCTS1 complex are summarized in Supplementary Data 1.

Multiple kinases can phosphorylate the DENR•MCTS1 complex in vitro

As a second parallel approach to study post-translational regulation of the DENR•MCTS1 complex, we aimed to discover kinases that phosphorylate and regulate this complex. An *in vitro* kinase assay with 245 mammalian Ser/Thr kinases and recombinant DENR•MCTS1 protein complex identified 50 kinases with the capacity to phosphorylate DENR•MCTS1 *in vitro*, yielding a signal >3-fold above the background signal caused by kinase autophosphorylation (Supplementary Data 2). We further tested whether any of these kinases regulates DENR•MCTS1 activity *in vivo* by knocking down their expression and assaying stuORF reporter activity in two cell lines: HeLa and MCF7 (Suppl. Figure 9a-b). While only PRKG1 and PRKCQ significantly dropped the stuORF activity in both cell lines, we observed that many of the top hits in both cell lines belonged to the family of Polo-like (PLK1, PLK2, PLK3, PLK4) or Hippo (STK3/MST2, STK4/MST1) kinases (Suppl. Figure 9a-b). To identify

the sites in DENR and/or MCTS1 that can be phosphorylated by these kinases, we performed radioactive *in vitro* kinase assays using a panel of mutant DENR and MCTS1 proteins. Since kinases often phosphorylate multiple residues on a target protein, mutating single residues often gives ambiguous results. Hence we mutated to alanine all serines and threonines in DENR and MCTS1 that were observed or predicted to be phosphorylated, and then singly back-mutated individual residues to serine or threonine to reveal phosphorylation sites. This approach showed that *in vitro* PLK3 phosphorylates DENR predominantly on Ser189 (Suppl. Figure 9c). In some cases, phosphorylation by a Polo-like kinase, however, relies on a priming phosphorylation by a cyclin-dependent kinase (most commonly CDK1) [3] , which is absent in protein complexes purified from a bacterial expression system. Therefore, it is possible that there are additional PLK phosphorylation sites in the DENR•MCTS1 complex which we did not identify. Apart from PLK3, we found that STK3 phosphorylates MCTS1 on Thr81 and Thr179, which lie within STK consensus motifs (Suppl. Figure 9d). Also STK4 was able to phosphorylate MCTS1 *in vitro* (data not shown). Several results, however, precluded us from following up on these kinases. Regarding DENR Ser189, we were unable to produce a working phospho-antibody for this site, and mutation of this site to alanine did not show a loss in stuORF activity upon DNA damage, when PLK3 should be active (data not shown). Similarly, expression of non-phosphorylatable mutants MCTS1 Thr81Ala or Thr179Ala did not have a clear impact on stuORF activity (Suppl. Figure 8b, data not shown). Nonetheless, we present these data here because they may be useful or relevant in the future when studying other cell lines or cellular conditions.

Supplementary Figure 1

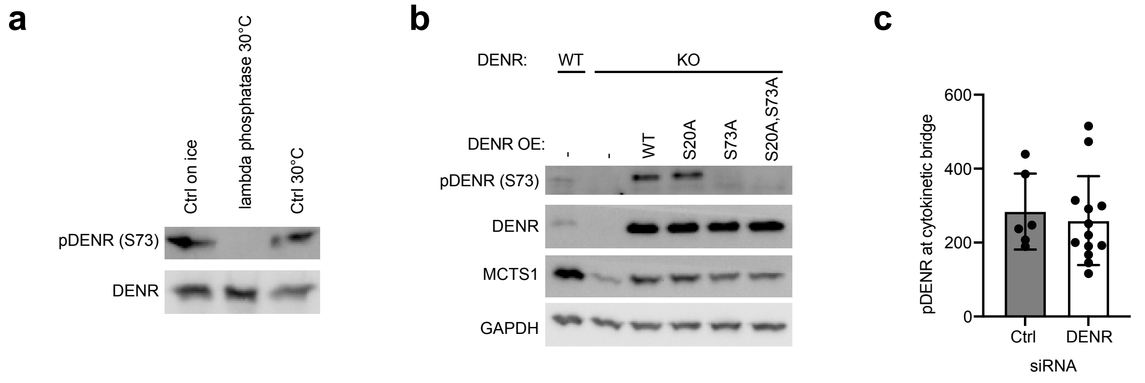

Supplementary Figure 1: DENR is phosphorylated at Ser73

(a-b) Our custom-made phospho-DENR(Ser73) antibody specifically detects DENR when phosphorylated on Ser73. (a) The signal is phospho-specific, demonstrated by dephosphoylating HeLa cell lysates with lambda phosphatase. Samples were incubated on ice or at 30°C with or without lambda phosphatase for 30 minutes and analysed by Western Blot. (b) The signal is specific for phosphorylation on Ser73. HeLa $DENR^{WT}$ or $DENR^{KO}$ cells were transfected with the indicated constructs, collected and analysed by Western blot. Note that in this experiment the overexpressed DENR levels are higher than the endogenous DENR levels in lane 1.

(c) The signal observed at the cytokinetic bridge upon staining cells with pDENR antibody is not specific, demonstrated in asynchronous U2OS cells after transfection with either control (grey) or DENR (white) siRNA, where only signal on the cytokinetic bridge was quantified in n=6 (Ctrl siRNA) or n=13 (DENR siRNA) independent mitotic cells. Standard deviations are displayed as error bars.

Supplementary Figure 2

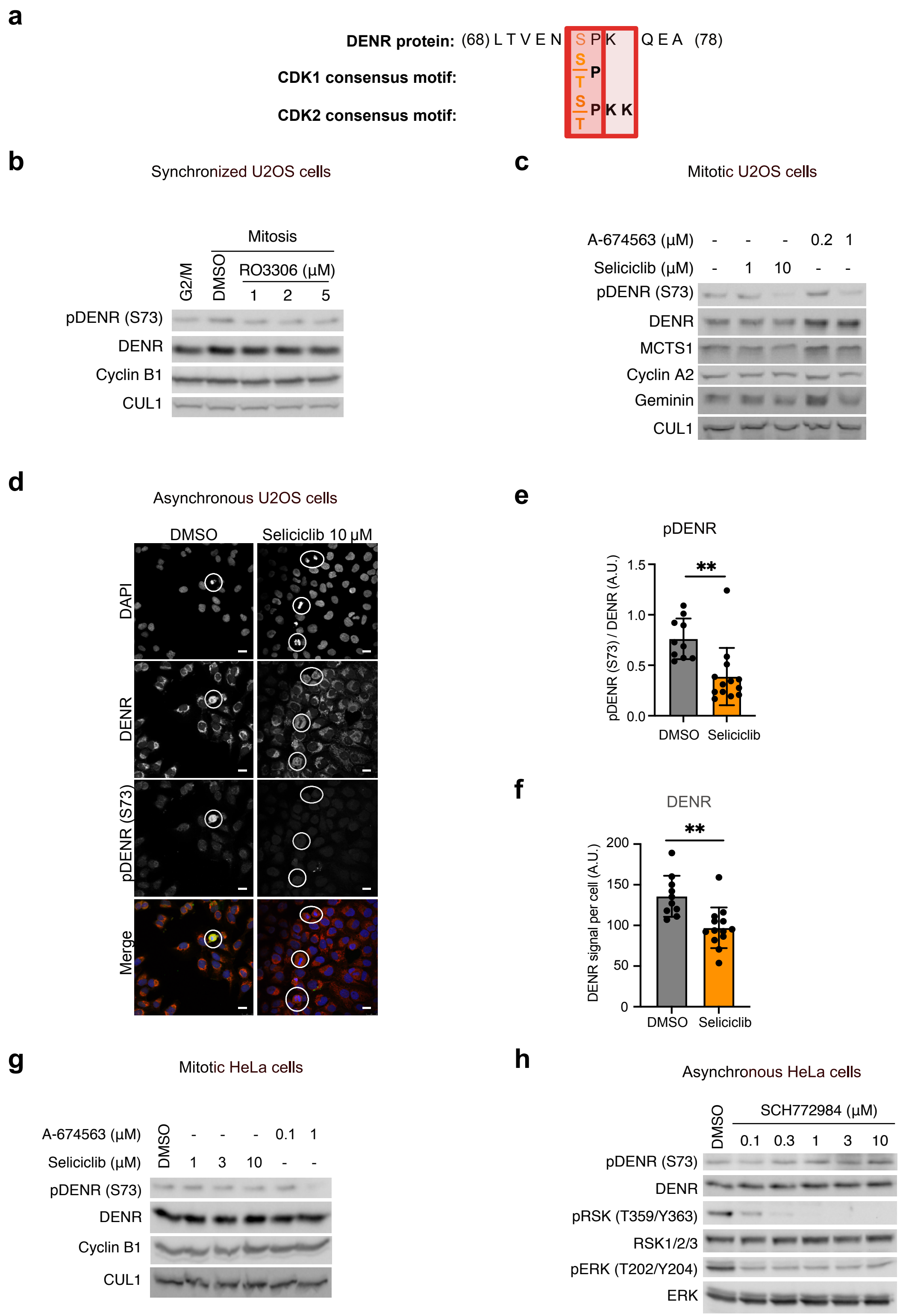

Supplementary Figure 2: CDK1 and CDK2 phosphorylate DENR at Ser73
(a) DENR Ser73 (orange) resides within canonical CDK consensus motifs (red boxes).

(figure legend continues on next page)

(b) Mitotic DENR phosphorylation in U2OS cells is reduced upon CDK1 inhibition. U2OS cells were synchronized in G2 phase, followed by release and – 30 minutes later (“G2/M”, lane 1) –either DMSO or CDK1 inhibitor RO3306 were added for 45 minutes.

(c) DENR (S73) phosphorylation in mitotic U2OS cells shows a dose-dependent decrease in response to seliciclib or A-674563 treatment, that was added for four hours during entry to mitosis after release from thymidine-induced S phase arrest.

(d-f) Exposure of asynchronous U2OS cells to seliciclib 10μM for one hour reduces pDENR(S73), detected by immunostaining. (d) Representative images with mitotic cells circled. Scale bars indicate 10μM. (e) pDENR(S73) and (f) total DENR signal per cell for mitotic cells – identified by DAPI stain - were quantified using ImageJ. Displayed are means and standard deviations as error bars. Unpaired t-test was performed on measurements from n=10 (DMSO, grey) or n=13 (seliciclib, orange) inidividual cells with **p=0.002 (e) or **p=0.001 (f).

(g) DENR phosphorylation is only mildly reduced upon Cdk2 inhibition in HeLa cells. Mitotic DENR phosphorylation is assessed by synchronization of HeLa cells in S phase, followed by release and – 6 hours later – addition of either DMSO or the indicated doses of Seliciclib or A674563 for three hours. Representative Western Blots of n=2 independent biological replicates are shown.

(h) DENR Ser73 phosphorylation is not reduced upon ERK1/2 inhibition, assessed via Western Blot analysis of asynchronous HeLa cells exposed to increasing doses of the ERK1/2 inhibitor SCH772984 for 1 hour. Phosphorylation of the ERK substrate RSK and ERK autophosphorylation are used as positive controls for drug efficacy. Representative Western Blots from n=3 independent biological replicates are shown.

(figures continue on next page)

Supplementary Figure 3

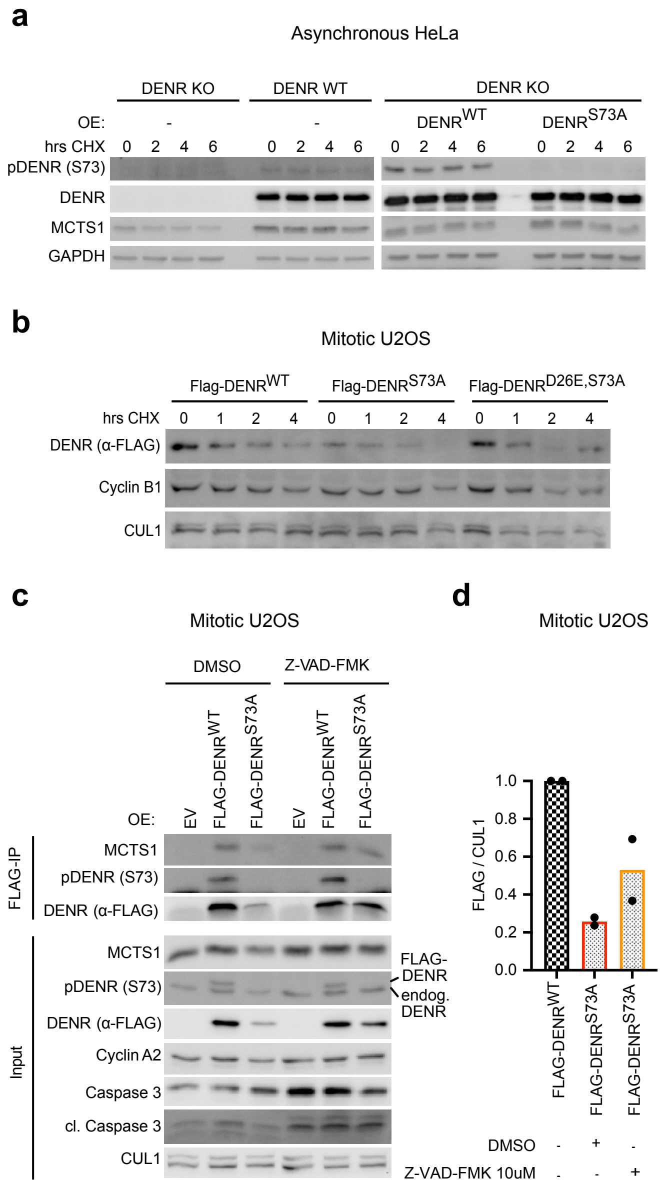

Supplementary Figure 3: DENR^{S73A} is caspase-cleaved at position D26 and instable in mitosis

(a) Both DENR^{WT} and DENR^{S73A} are comparably stable in interphase cells. Asynchronous DENR^{WT} or DENR^{KO} HeLa cells transfected to re-express either DENR^{WT} or DENR^{S73A} were treated with cycloheximide at a concentration of 100µg/ml for the indicated times and then lysed for immunoblotting.

(b) Mitotic stability of DENR^{S73A} is partially re-established by introducing the cleavage site mutation D26E. Cells were transfected to express either version of DENR, then synchronized in mitosis, harvested by shake-off, and finally exposed to cycloheximide for the indicated times and analysis by Western Blot.

(c-d) Both stability of DENR^{S73A} and its binding to MCTS1 in mitotic U2OS cells are partially re-established by addition of the pan caspase inhibitor Z-VAD-FMK (lanes 4-6, orange line in d) vs. DMSO (lanes 1-3, red line in d). Immunoprecipitation of overexpressed FLAG-DENR^{WT} (checked in d) or FLAG-DENR^{S73A} (finely checked in d) from mitotically synchronized U2OS cells is shown, n=2 independent biological replicates were performed and quantification of the FLAG-DENR signal, normalized to CUL1 is shown in (d).

Supplementary Figure 4

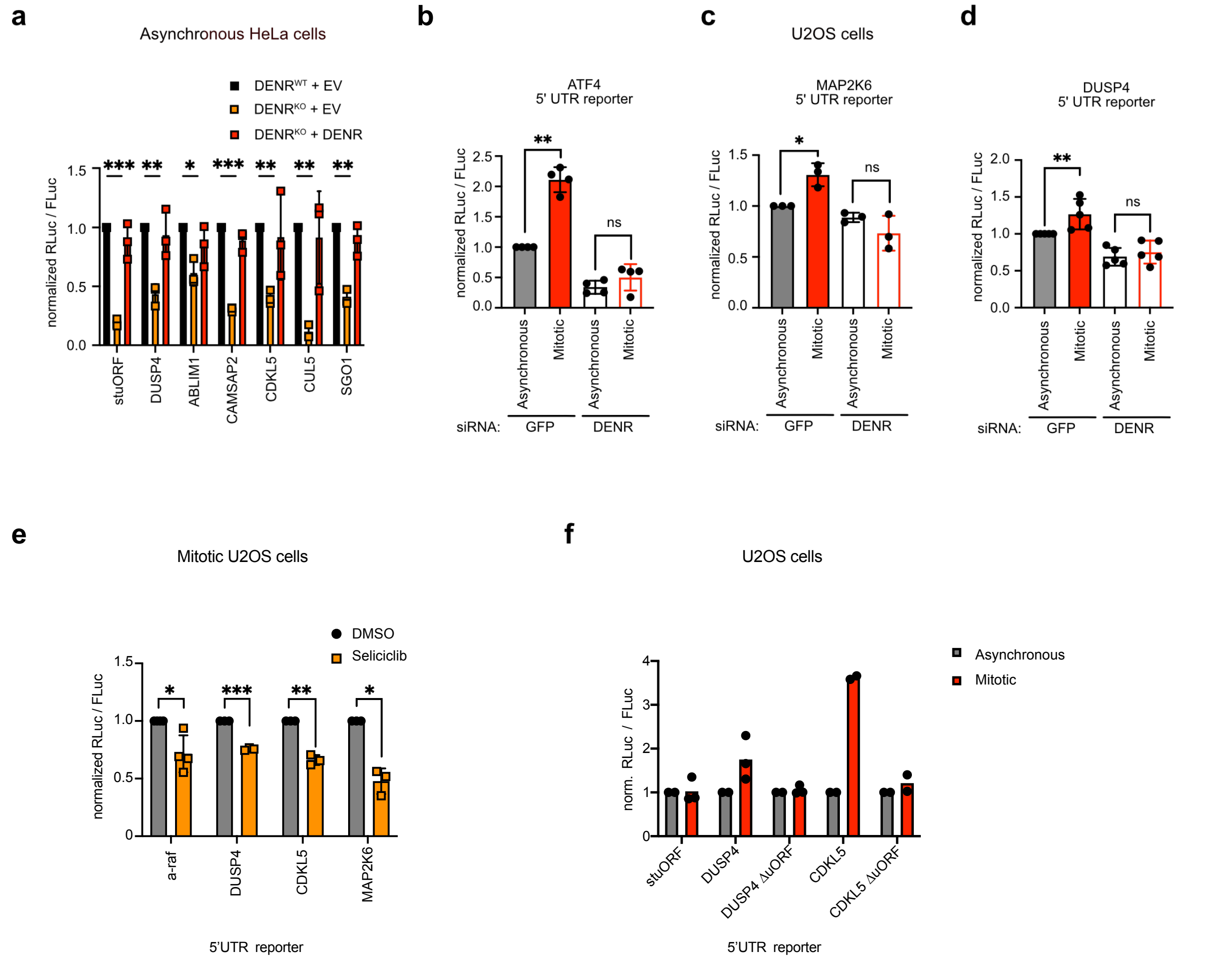

Supplementary Figure 4: DENR-dependent mitotic translation of reporters carrying the 5'UTRs of DENR target mRNAs

(a) Validation of mitotic DENR target genes by showing that their 5'UTRs impart DENR-dependence to a luciferase reporter. Luciferase activity of reporter constructs carrying the 5'UTR of the indicated genes decreases in DENR knockout (orange) versus DENR wildtype (black) cells and is rescued by reconstituting these cells to express DENR protein (red). Means were normalized to the $DENR^{WT}$ + EV condition for each target genes. One sample t-tests were applied to n=3 independent biological replicates with ***p=0.0005 (stuORF), **p=0.008 (DUSP4), *p=0.03 (ABLIM1), ***p=0.0002 (CAMSAP2), **p=0.003 (CDKL5), **p=0.001 (CUL5), **p=0.004 (SGO1).

(b-d) Translation of reporter constructs carrying 5'UTRs of DENR target genes increases in mitosis in a DENR-dependent manner. U2OS cells treated with GFP siRNA (filled) or a pool of three different DENR siRNAs (empty), re-plated 48-72 hours later, transfected with the indicated reporters and then synchronized in mitosis (red) or not synchronized (grey), show induction of mitotic translation in control but not in DENR knockdown cells. Displayed are means from independent biological replicates. One sample t-tests were applied to n independent biological replicates with (b) n=5, **p=0.002, (c) n=3, *p=0.04, (d) n=4, **p=0.005. Unpaired t-tests were applied with (b) n=5, ns p=0.23; (c) n=3, ns p=0.21; (d) n=4,ns p=0.49.

(figure legend continues on next page)

(e) Mitotic translation of DENR targets is inhibited upon CDK2 inhibition. U2OS cells were synchronized in mitosis using a double thymidine block, during the thymidine block transfected with indicated reporters, and after release from thymidine synchronized in mitosis for 13 hours using nocodazole. DMSO (grey) or seliciclib (orange) were added for the last 4 hours before analysis. After normalization to the DMSO condition one sample t-tests were performed from n=4 (a-raf) or n=3 (all others) biological replicates with *p=0.04 (a-raf), ***p=0.0007 (DUSP4), **p=0.005 (CDKL5) and *p=0.01 (MAP2K6). Means are displayed.

(f) Mutation of the ATGs of all uORFs within the DUSP4 or CDKL5 5'UTR abrogate induction of reporter mitotic translation. U2OS cells, that were transfected with the indicated 5'UTR-reporters were mitotically synchronized using sequential thymidine-STLC (red) or left unsynchronized (grey). Data are from n=3 (stuORF, DUSP4) or n=2 (CDKL5) independent biological replicates are shown.

In all subfigures error bars indicate standard deviations.

(figures continue on next page)

Supplementary Figure 5

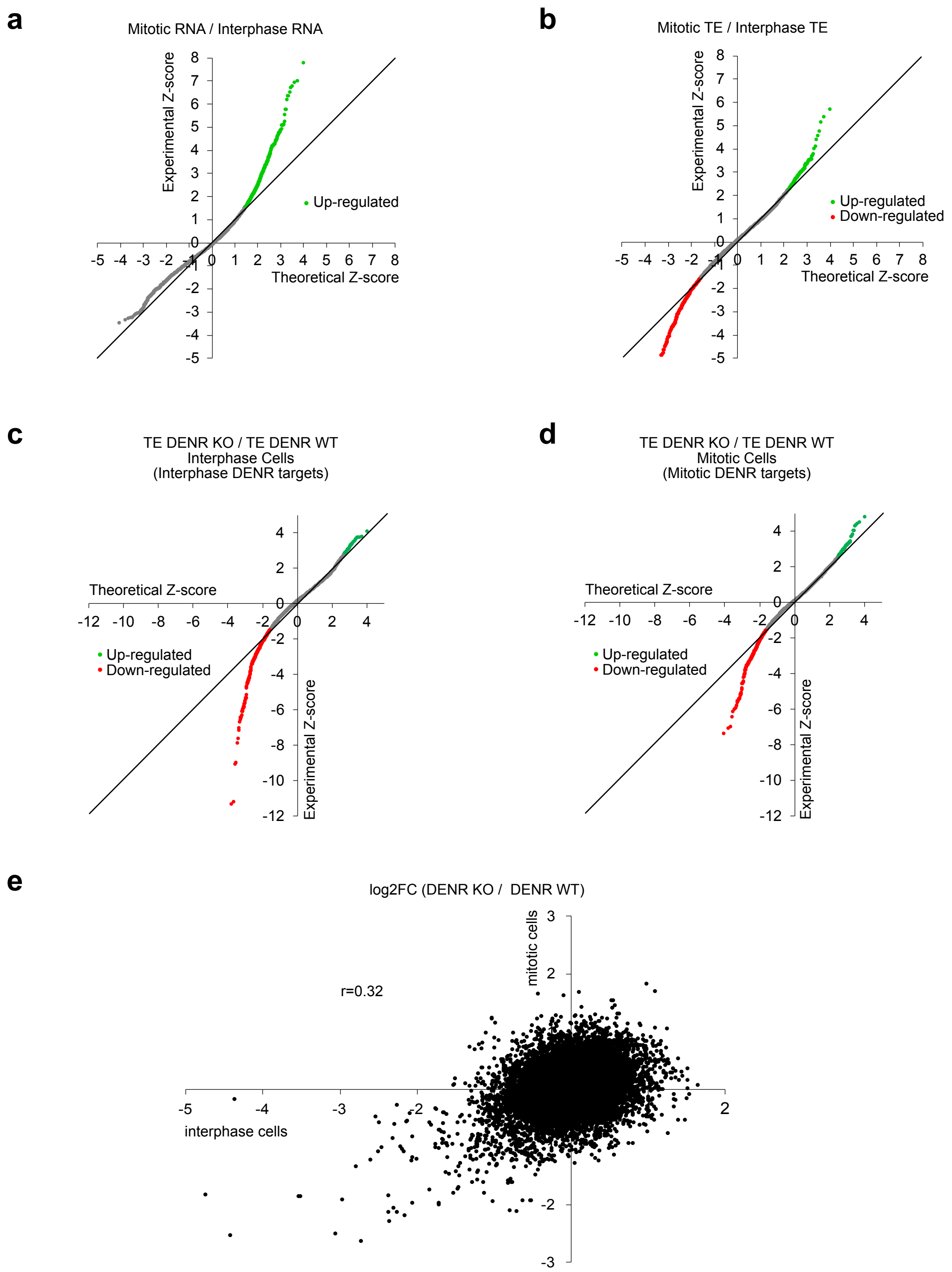

Supplementary Figure 5: Ribosome profiling to identify mitotic DENR target transcripts
Mitotic or interphase HeLa DENR^{WT}, DENR$^{KO_clone\ 2.11}$ or DENR$^{KO_clone\ 3.42}$ cells were analysed by RNA-Seq and RiboSeq. A Z-vs-Z analysis (comparing observed Z-scores to Z-scores expected theoretically from a normal distribution) identifies transcripts that have (a) altered mRNA levels in mitotic wildtype cells compared to interphase cells, (b) altered translation efficiency (RiboSeq normalized to RNA-Seq) in mitotic wildtype cells compared to interphase cells), or altered translation efficiency in DENR knockout (c) interphase or (d) mitotic cells compared to equivalent wildtype cells. (e) Comparison of the log2(FC) in TE in DENR knockout cells versus wildtype cells for asynchronous cells (x-axis) or mitotic cells (y-axis) reveals a good correlation. Throughout the figure upregulated genes/transcripts are indicated in green, unchanged genes/transcripts in grey, downregulated genes/transcripts in red.

Supplementary Figure 6

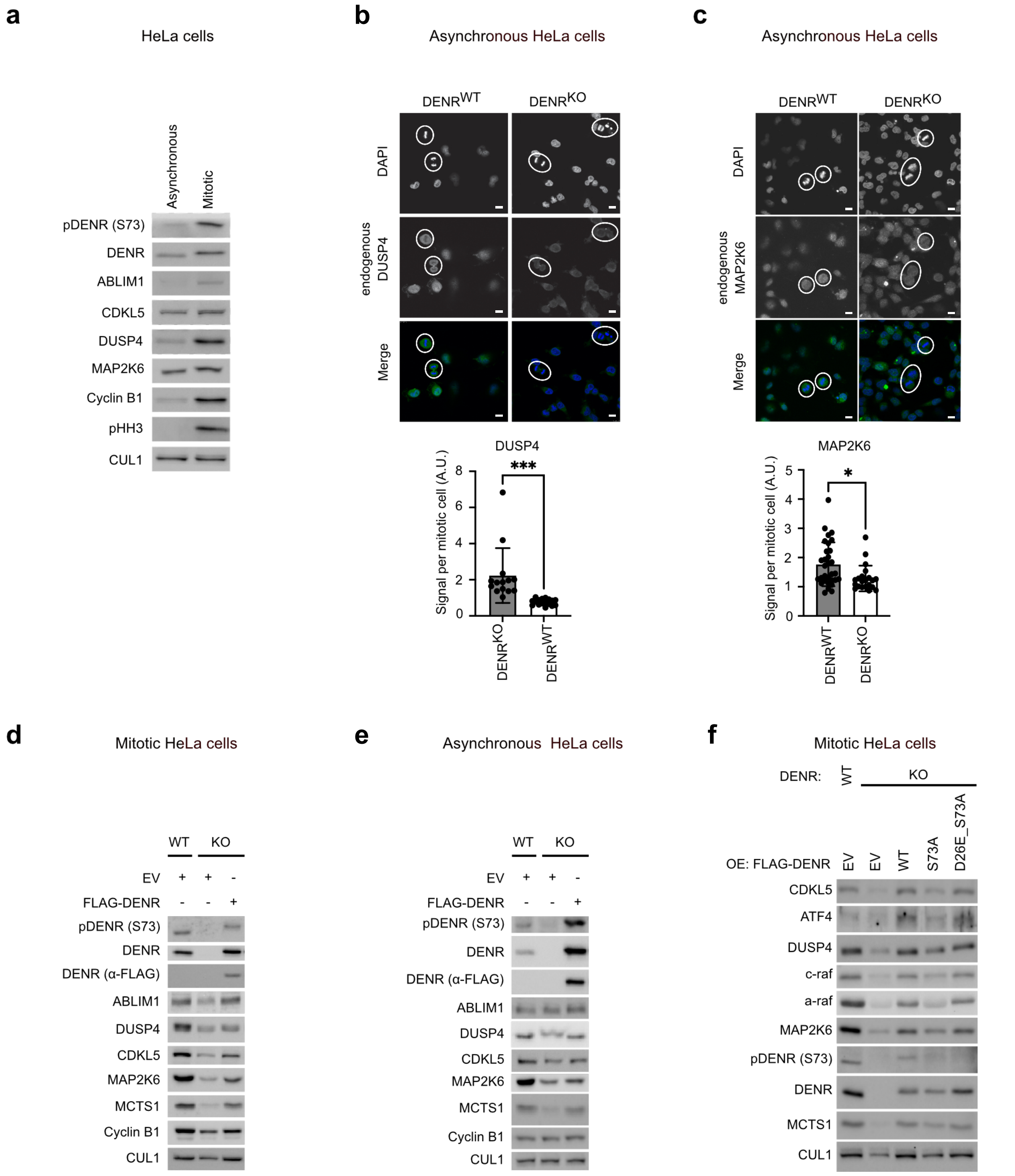

Supplementary Figure 6: DENR-dependent translation of mitotic target proteins

(a) Protein levels of DENR target genes increase in mitotic HeLa cells synchronized using sequential thymidine-STLC treatment compared to asynchronous HeLa cells. Representative Western Blots from n=3 independent biological replicates are shown.

(figure legend continues on next page)

(b-c) Endogenous levels of DENR target proteins DUSP4 (b) or MAP2K6 (c) are reduced in mitotic DENR knockout cells compared to mitotic control cells. Unsynchronized HeLa $DENR^{WT}$ (grey) or $DENR^{KO}$ (white) cells immunostained for DUSP4 or MAP2K6. For quantification, (b) n=14 $DENR^{WT}$ and 17 $DENR^{KO}$ mitotic cells, (c) n=31 $DENR^{WT}$ and 21 $DENR^{KO}$ mitotic cells. Means of signal intensities and standard deviations as error bars are displayed. Unpaired t-test was applied with ***p=0.0004 (b) and *p=0.01 (c). Scale bars indicate 10μM.

(d-e) Protein levels of endogenous DENR targets are more strongly reduced upon DENR loss-of-function in mitotic cells (d) than asynchronous cells (e). HeLa $DENR^{WT}$ or $DENR^{KO}$ cells, that were transfected with an empty expression vector (EV) or a FLAG-$DENR^{WT}$ construct and either mitotically enriched by a nine hour release from thymidine (d) or not (e). Loss of DENR causes a drop in target proteins that is rescued with DENR reexpression. Representative images of n=2 independent biological replicates are shown.

(f) Same samples as shown in main Figure 4j, but here including $DENR^{WT}$ and $DENR^{KO}$ cells. Loading here is not optimal (see CUL1) due to the reduced viability of $DENR^{KO}$ and $DENR^{KO}$+DENRS73A cells. Representative Western Blots of n=2 independent biological replicates are shown.

(figures continue on next page)

Supplementary Figure 7

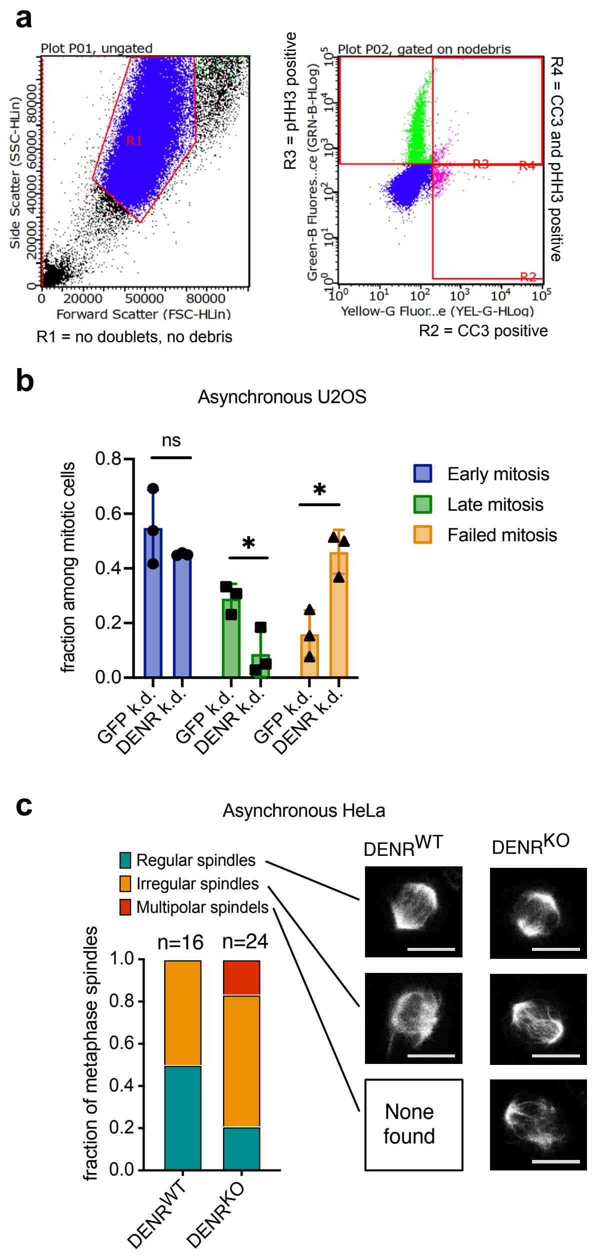

Supplementary Figure 7: DENR loss of function causes aberrant mitoses and mitotic cell death.

(a) Gating strategy for flow cytometry of unsynchronized HeLa cells underlying analysis in Figure 5a,b: in a first step, cell debris and doublets were gated out. In a second step, fractions of CC3, pHH3 (Figure 5a) or double positive (Figure 5b) cells were measured.

(b) U2OS cells were transfected with either Ctrl or siRNA against DENR and otherwise left untreated. Confocal microscopy and quantification of early (prophase, prometaphase, metaphase; blue), late (anaphase, telophase; green) or failed (aberrant mitosis, mitotic blebs; orange) mitoses confirms increased mitotic failure after DENR loss of function. Data are from n=3 independent biological replicates and unpaired t-tests were performed with p(early)=0.3, *p(late)=0.048 and *p(failed)=0.049. Error bars indicate standard deviations.

(c) Asynchronous HeLa DENR^{WT} or DENR^{KO} cells were immunostained for γ-tubulin and spindle morphologies (regular spindles: green, irregular spindles: orange; multipolas spindles: red) from n=16 (DENR^{WT}) or n=24 (DENR^{KO}) independent mitotic cells were counted and displayed. Scale bars indicate 10µM

Supplementary Figure 8

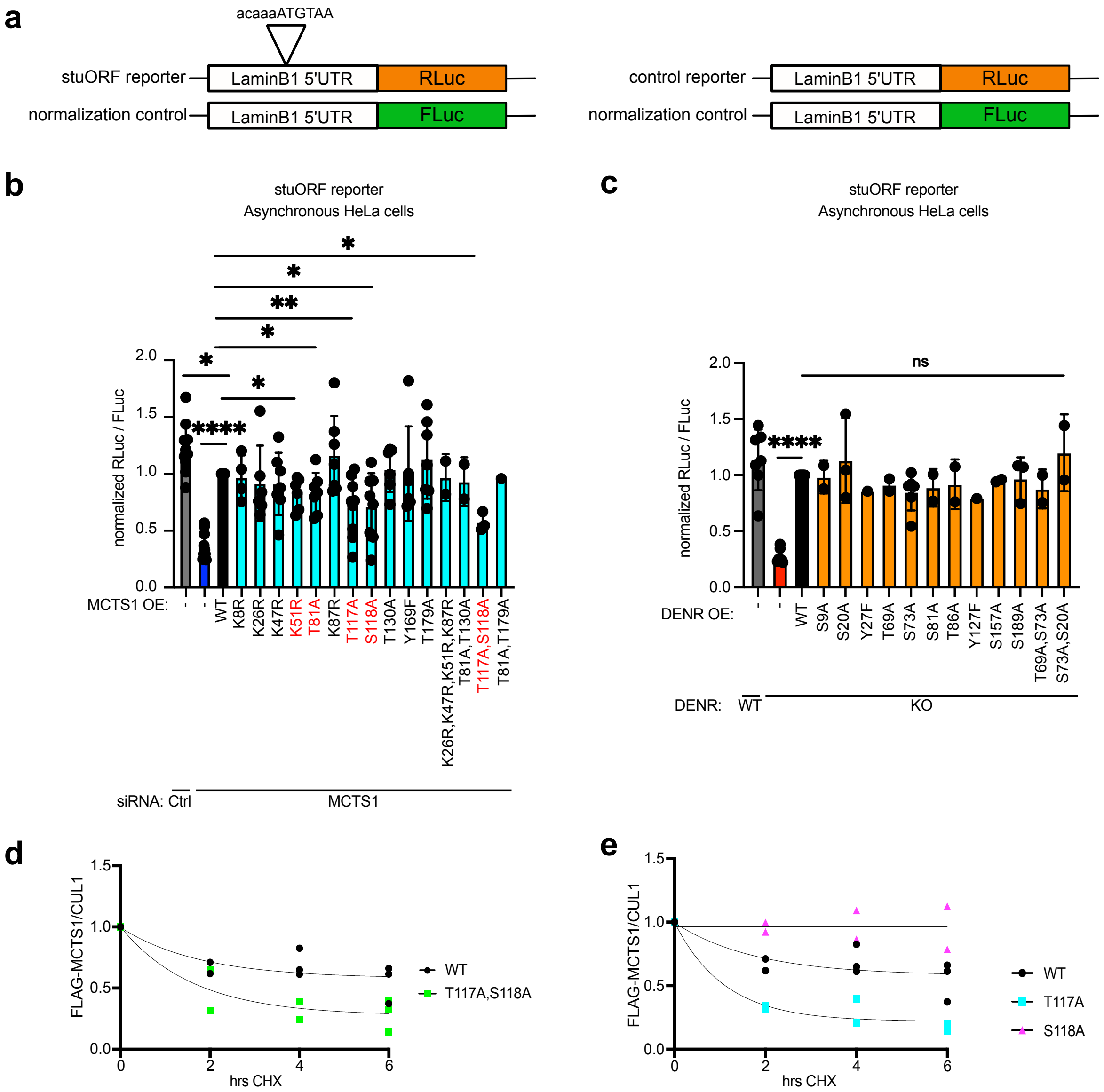

Supplementary Figure 8: Testing the functional consequence of point mutations that abolish DENR•MCTS post-translational modifications

(a) Diagram of reporter constructs used to test stuORF-dependent translation. Either cells were transfected with a LaminB1 5'UTR-renilla luciferase (RLuc) reporter into which an upstream Open Reading Frame with a strong Kozak sequence (stuORF) was inserted, or with a negative control LaminB1-RLuc reporter lacking the stuORF, both together with a firefly luciferase (FLuc) normalization control. For each well, RLuc/FLuc ratios were calculated. The stuORF reporter ratio was normalized to the negative control ratio to control for density and siRNA-specific effects.

(figure legend continues on next page)

(b) MCTS1 activity tested by reconstituting MCTS1 knockdown cells with various MCTS1 mutants. GFP- (first lane) or MCTS1- (all following lanes) siRNA-treated HeLa cells were transfected with empty plasmid control or the indicated siRNA-insensitive MCTS1 constructs as well as RLuc and FLuc reporter plasmids indicated in (a), and then analysed by luciferase. Values are normalized to MCTS1^{WT} reconstitution from n=1 (T81A,T179A), n=2 (K26R,K47R,K51R,K87R; T81A,T130A), n=3 (T117A,S118A), n=4 (K8R), n=6 (K26R; K51R; T130A; Y169F), n=7 (K47R; T81A; K87R; T179A), n=8 (S118A), n=9 (T117A) or n=10 (Ctrl knockdown; MCTS1 knockdown; WT) independent biological replicates are shown. One sample t-tests were applied with *p=0.01 (Ctrl knockdown), ****p<0.0001 (MCTS1 knockdown), *p=0.03 (K51R), *p=0.046 (T81A), **p=0.01 (T117A), *p=0.03 (S118A), *p=0.01 (T117A_S118A).

(c) DENR activity tested by reconstituting DENR knockout cells with various DENR mutants. HeLa DENR^{WT} (first lane) or DENR^{KO} (all following lanes) cells were transfected with empty expression vector (first two lanes) or one of the indicated DENR constructs as well as the luciferase constructs described in (a) and analysed by luciferase assay. Values are normalized to DENR^{WT} reconstitution from n=1 (Y27F; Y127F), n=2 (S9A; T69A; S81A; T86A; S157A; T69A,S73A; S20A,S73A), n=3 (S20A; S189A), n=6 (S73A) or n=7 (DENR_WT; DENR_KO; WT) independent biological replicates. One sample t-test was applied with ****p (DENR^{KO} + EV)<0.0001

In (b) and (c) means and standard deviations as error bars are shown.

(d-e) FLAG-MCTS1^{T117A} (h) and FLAG-MCTS1^{T117A,S118A} (g) are less stable, but FLAG-MCTS1^{S118A} (h) is more stable than FLAG-MCTS^{WT}. HeLa cells were transfected to express the different versions of MCTS1, exposed to cycloheximide and analysed by Western Blot. Quantification of FLAG signal normalized to CUL1 from n=3 independent biological replicates.

(figures continue on next page)

Supplementary Figure 9

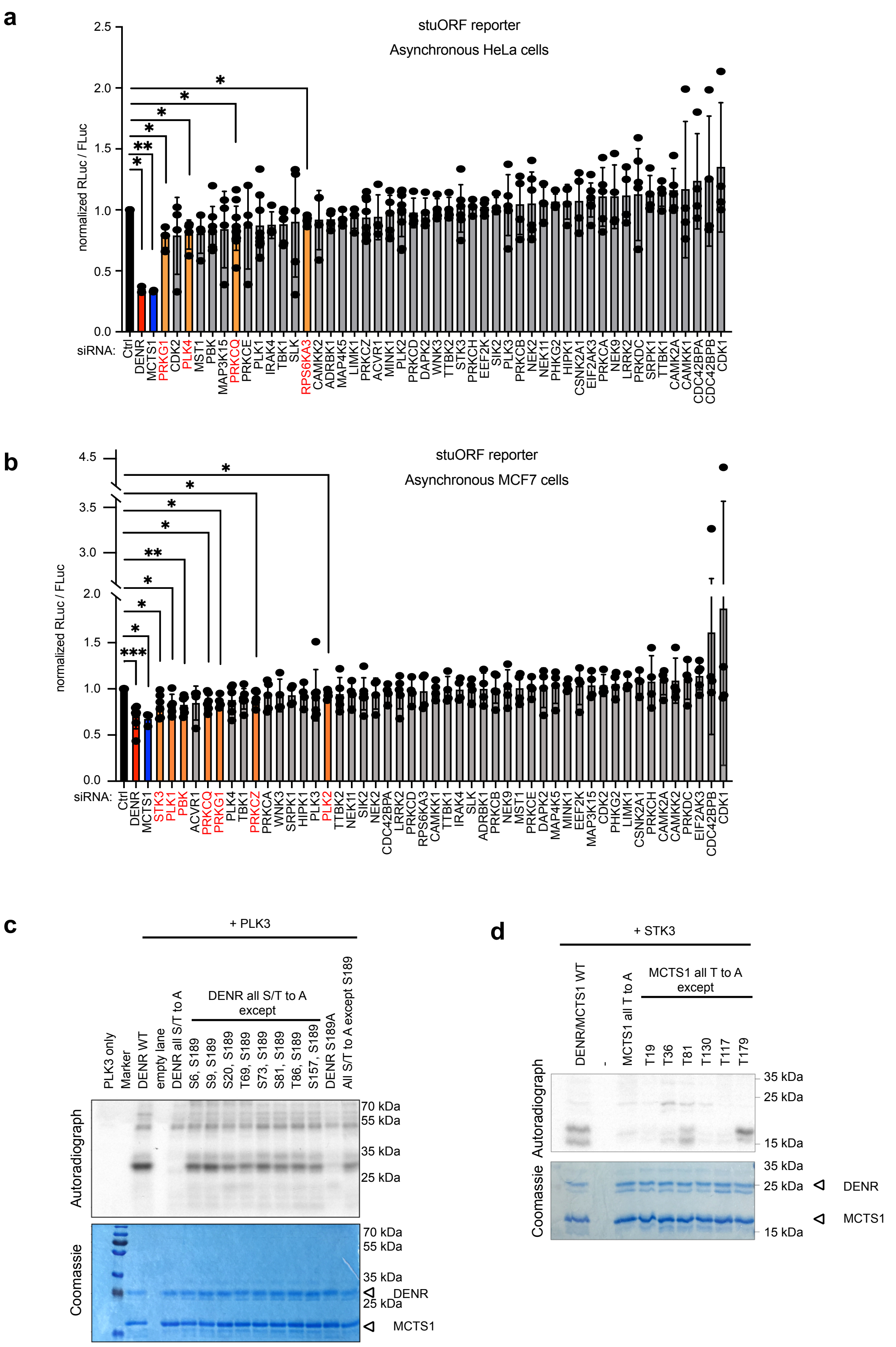

Supplementary Figure 9: Kinases acting upon the DENR•MCTS1 complex

(figure legend continues on next page)

(a-b) Several kinases significantly influence translation of a stuORF reporter in (a) HeLa cells or (b) MCF7 cells. Cells were transfected with siRNAs against GFP, DENR, MCTS1 or one of the kinases that are able to phosphorylate the DENR•MCTS1 complex *in vitro*, then transfected with stuORF-RLuc or Ctrl-FLuc reporter plasmids three days later and analysed by luciferase assay. Values are normalized to Ctrl knockdown and means and standard deviations as error bars (a) from n=2 (DENR knockdown, MCTS1 knockdown), n=5 (SLK, MINK1, PLK3, PRKCA, CAMK2A), n=6 (TBK1, STK3, NEK2, EIF2AK3, PRKDC), n=7 (PBK), n=8 (PRKCZ), n=9 (PLK1, PLK2), n=10 (Ctrl knockdown, PRKCQ) or n=4 (all others) and (b) from n=3 (MCTS1), n=5 (PLK1, PRKCA, PLK2, TTBK2, LRRK2, PRKCB, EIF2AK3), n=6 (STK3, PBK, PRKCQ, PLK4, TBK1, PRKCZ, SIK2), n=7 (DENR, PLK3), n=8 (Ctrl knockdown) or n=4 (all others) independent biological replicates are shown. One sample t-tests (a) *p(DENR)=0.02, **p(MCTS1)=0.005, *p(PRGK1)=0.01, *p(PLK4)=0.04, *p(PRKCQ)=0.03, *p(RPS6AK3)=0.02; (b) ***p(DENR)=0.0008, *p(MCTS1)=0.02, *p(STK3)=0.02, *p(PLK1)=0.03, **p(PBK)=0.008, *p(PRKCQ)=0.02, *p(PRKG1)=0.03, *p(PRKCZ)=0.04, *p(PLK2)=0.03

(c) PLK3 phosphorylates DENR at Ser189 *in vitro*. Recombinant DENR•MCTS1 complex or one of its mutants were purified from bacteria, analysed by Coomassie Brillant Blue staining, exposed to active recombinant PLK3 and ^{32}P in an *in vitro* kinase assay, and then analysed. In the “DENR all S/T to A” mutant the following sites are mutated within DENR: S6A, S9A, S20A, T69A, S73A, S81A, T86A, S157A, and S189A.

(d) STK3 phosphorylates MCTS1 at threonines 81 and 179 *in vitro*. Recombinant DENR•MCTS1 complex or one of its mutants were purified from bacteria, analysed by Coomassie Brillant Blue staining, exposed to active recombinant STK4 and ^{32}P in an *in vitro* kinase assay, and then analysed. In the “all T to A” mutant the following sites are mutated within MCTS1: T19A, T36A, T81A, T117A, T130A, T179A.

<u>3) Supplementary References</u>

1. Schleich S*, et al.* DENR-MCT-1 promotes translation re-initiation downstream of uORFs to control tissue growth. *Nature* **512**, 208-212 (2014).

2. Schleich S, Acevedo JM, Clemm von Hohenberg K, Teleman AA. Identification of transcripts with short stuORFs as targets for DENR*MCTS1-dependent translation in human cells. *Scientific reports* **7**, 3722 (2017).

3. Singh P*, et al.* BUB1 and CENP-U, Primed by CDK1, Are the Main PLK1 Kinetochore Receptors in Mitosis. *Mol Cell* **81**, 67-87 e69 (2021).
