# Supplementary material for: Cyclin B/CDK1 and Cyclin A/CDK2 phosphorylate DENR to promote mitotic protein translation and faithful cell division
Source: Nat Commun. 2022 Feb 3;13:668. doi: 10.1038/s41467-022-28265-0 (PMC8813921; doi:10.1038/s41467-022-28265-0)
Supplement: Supplementary file 3 — Description of Additional Supplementary Files [file 41467_2022_28265_MOESM3_ESM.pdf]

Description of Additional Supplementary Files

File name: Supplementary Data 1

Description: Summary of data obtained for phosphorylation sites in the DENR/MCTS1 complex

File name: Supplementary Data 2

Description: In vitro kinase assay of Ser/Thr kinases on DENR•MCTS1 complex

File name: Supplementary Data 3

Description: DENR targets and their roles in mitosis

File name: Supplementary Data 4

Description: Data matrix for RNAseq and Riboseq of mitotic and interphase DENR-WT and DENRKO cells (2 independent KO clones)

File name: Supplementary Data 5

Description: Transcripts and genes with significantly increased mRNA levels in mitotic cells compared to interphase cells

File name: Supplementary Data 6

Description: Transcripts and genes with significantly increased or decreased translation efficiency (TE) in mitotic cells compared to interphase cells

File name: Supplementary Data 7

Description: DENR targets in Interphase and Mitosis

File name: Supplementary Data 8

Description: Sequences of oligos used for cloning

File name: Supplementary Data 9

Description: siRNA catalog numbers and sequences
