# Supplementary material for: Cyclin B/CDK1 and Cyclin A/CDK2 phosphorylate DENR to promote mitotic protein translation and faithful cell division
Source: Nat Commun. 2022 Feb 3;13:668. doi: 10.1038/s41467-022-28265-0 (PMC8813921; doi:10.1038/s41467-022-28265-0)
Supplement: Supplementary file 13 — Reporting Summary [file 41467_2022_28265_MOESM13_ESM.pdf]

nature research

Corresponding author(s): Aurelio A. Teleman

Last updated by author(s): Dec 6, 2021

Reporting Summary

Nature Research wishes to improve the reproducibility of the work that we publish. This form provides structure for consistency and transparency in reporting. For further information on Nature Research policies, see our Editorial Policies and the Editorial Policy Checklist.

Statistics

For all statistical analyses, confirm that the following items are present in the figure legend, table legend, main text, or Methods section.

n/a	Confirmed	
☐	☒	The exact sample size (*n*) for each experimental group/condition, given as a discrete number and unit of measurement
☐	☒	A statement on whether measurements were taken from distinct samples or whether the same sample was measured repeatedly
☐	☒	The statistical test(s) used AND whether they are one- or two-sided<br>*Only common tests should be described solely by name; describe more complex techniques in the Methods section.*
☒	☐	A description of all covariates tested
☐	☒	A description of any assumptions or corrections, such as tests of normality and adjustment for multiple comparisons
☐	☒	A full description of the statistical parameters including central tendency (e.g. means) or other basic estimates (e.g. regression coefficient) AND variation (e.g. standard deviation) or associated estimates of uncertainty (e.g. confidence intervals)
☐	☒	For null hypothesis testing, the test statistic (e.g. *F*, *t*, *r*) with confidence intervals, effect sizes, degrees of freedom and *P* value noted<br>*Give P values as exact values whenever suitable.*
☒	☐	For Bayesian analysis, information on the choice of priors and Markov chain Monte Carlo settings
☒	☐	For hierarchical and complex designs, identification of the appropriate level for tests and full reporting of outcomes
☒	☐	Estimates of effect sizes (e.g. Cohen's *d*, Pearson's *r*), indicating how they were calculated

Our web collection on statistics for biologists contains articles on many of the points above.

Software and code

Policy information about availability of computer code

Data collection	All software used is either freely available or commercially available:<br>ImageLab version 5.2.1<br>ImageJ 1.50i<br>Fiji/ImageJ version 2.1.0/1.53c<br>guava Soft 3.3
Data analysis	All software used is either freely available or commercially available:<br>ImageJ 1.50i<br>Fiji/ImageJ version 2.1.0/1.53c<br>GraphPad Prism version 9.0.0 (86)<br>Ape-A plasmid Editor version 2.0.49.10<br>guava Soft 3.3<br>bowtie2 version 2.3.4.2

For manuscripts utilizing custom algorithms or software that are central to the research but not yet described in published literature, software must be made available to editors and reviewers. We strongly encourage code deposition in a community repository (e.g. GitHub). See the Nature Research guidelines for submitting code & software for further information.

Data

Policy information about availability of data

All manuscripts must include a data availability statement. This statement should provide the following information, where applicable:

- Accession codes, unique identifiers, or web links for publicly available datasets
- A list of figures that have associated raw data
- A description of any restrictions on data availability

The manuscript includes the following Data Availability Statement:
The riboseq and RNA-seq datasets in this study have been deposited in the NCBI SRA database under accession code [http://www.ncbi.nlm.nih.gov/bioproject/768478]. The riboseq and RNASeq source data generated in this study are provided in the Supplementary Information.

The manuscript includes the following Code Availability Statment:
All custom software used in this study is available at GitHub: https://github.com/aurelioteleman/Teleman-Lab [https://github.com/aurelioteleman/Teleman-Lab] and in the Zenodo repository (10.5281/zenodo.5751288).

Field-specific reporting

Please select the one below that is the best fit for your research. If you are not sure, read the appropriate sections before making your selection.

☒ Life sciences ☐ Behavioural & social sciences ☐ Ecological, evolutionary & environmental sciences

For a reference copy of the document with all sections, see nature.com/documents/nr-reporting-summary-flat.pdf

Life sciences study design

All studies must disclose on these points even when the disclosure is negative.

Sample size	No statistical sample-size calculation was performed. Instead, experiments were performed with 3 biological replicates, which from experience is sufficient to assure that the selected examples are representative and reproducible. If the effect size was small, additional biological replicates were performed to ensure reproducibility. For each experiment we clearly state the number of independent biological replicates in the manuscript.
Data exclusions	In Figure 4b f, luciferase reporter for CDKL5 produced very low values; therefore, values below 40 (=background) were excluded from the analysis.
Replication	All technically sound attempts at replication of all experiments were successful. The number of independent biological replicates is clearly stated in the manuscript in every case.
Randomization	In all cases cells were randomly pipetted into the various wells of the experiment for the different treatments.
Blinding	For analysis of microscopy images in Figures 4 and 5 and Suppl. Figures 1, 2, 6 and 7 the investigator was not blinded to group allocation because the differences between the different groups were immediately obvious (cell morphology, mitotic accumulation, apoptotic cells). Also for the other experiments the investigators were not blinded to group allocation during data collection because the measurements are quantitative and difficult to influence by investigator bias.

Reporting for specific materials, systems and methods

We require information from authors about some types of materials, experimental systems and methods used in many studies. Here, indicate whether each material, system or method listed is relevant to your study. If you are not sure if a list item applies to your research, read the appropriate section before selecting a response.

Materials & experimental systems

n/a	Involved in the study	
☐	☒	Antibodies
☐	☒	Eukaryotic cell lines
☒	☐	Palaeontology and archaeology
☒	☐	Animals and other organisms
☒	☐	Human research participants
☒	☐	Clinical data
☒	☐	Dual use research of concern

Methods

n/a	Involved in the study	
☒	☐	ChIP-seq
☐	☒	Flow cytometry
☒	☐	MRI-based neuroimaging

Antibodies

Antibodies used

ABLIM1 (WB: 1:1000, IF: 1:500, rabbit, bethyl-biomol A302-237-T), β-Actin (1:5000, mouse, Sigma #A2228), ATF-4 (1:1000, rabbit, Cell Signalling #11815), Caspase 3 (1:1000, rabbit, Cell Signalling #9662), CDKL5 (WB: 1:1000, IF: 1:500, rabbit, abcam ab22453), CDT1 (1:1000, rabbit, Cell Signalling #8064), cleaved Caspase 3 (WB: 1:1000, flow cytometry 1:100, rabbit, Cell Signalling #9664), CUL1 (1:500, mouse, Invitrogen #32-2400), cyclin A2 (1:1000, rabbit, Cell Signalling #91500), cyclin B1 (1:1000, mouse, Cell Signaling #12231), cyclin E1 (1:1000, rabbit, Cell Signalling #20808), DENR (WB and IF: 1:2000, guinea pig, in-house production), pDENR_Ser73 (WB: 1:500, IF: 1:200, rabbit, custom-made by innovagen AB, Lund, Sweden), DUSP4 (WB: 1:1000, IF: 1:500, rabbit, abcam ab216576), FLAG (1:1000, rabbit, Sigma #F7425), FLAG-M2 (1:1000, mouse, Sigma #F3165), gamma-Tubulin (1:1000, Abcam, #ab27074), GAPDH (1:2000, rabbit, Cell Signallling #2118), Geminin (1:1000, rabbit, Cell Signalling #52508), pHH3 (Serine 10) (for WB: 1:500, rabbit, Cell Signaling #9701; for flow cytometry: 1:100, mouse, Cell Signalling #9706S), MAP2K6 (WB: 1:1000, IF: 1:500, rabbit, Cell Signalling #8550), MCTS1 (1:1000, guinea pig, in-house production), a-raf (1:1000, rabbit, Cell Signaling #4432), c-raf (1:1000, rabbit, Cell Signaling #9422), ERK1/2 (1:1000, rabbit, Cell Signaling #4695), GST-HRP conjugate (1:5000, goat, GE Healthcare #RPN1236), pERK1/2 (T202/Y204) (1:1000, rabbit, Cell Signaling #4370), p-p90RSK (T359/Y363) (1:1000, rabbit, Cell Signaling #9344),RSK1/2/3 (1:1000, rabbit, Cell Signaling #14813).

Validation

ABLIM1 (WB: 1:1000, IF: 1:500, rabbit, bethyl-biomol A302-237-T): WB manufacturer's website and siRNA-mediated knockdown, IF siRNA-mediated knockdown
β-Actin (1:5000, mouse, Sigma #A2228): WB manufacturer's website
ATF-4 (1:1000, rabbit, Cell Signalling #11815): WB and IF manufacturer's website
Caspase 3 (1:1000, rabbit, Cell Signalling #9662): WB manufacturer's website
CDKL5 (WB: 1:1000, IF: 1:500, rabbit, abcam ab22453): WB manufacturer's website and siRNA-mediated knockdown, IF siRNA-mediated knockdown
CDT1 (1:1000, rabbit, Cell Signalling #8064): WB manufacturer's website
cleaved Caspase 3 (WB: 1:1000, flow cytometry 1:100, rabbit, Cell Signalling #9664): WB and flow cytometry manufacturer's website
CUL1 (1:500, mouse, Invitrogen #32-2400): WB manufacturer's website
cyclin A2 (1:1000, rabbit, Cell Signalling #91500): WB manufacturer's website
cyclin B1 (1:1000, mouse, Cell Signaling #12231): WB manufacturer's website
cyclin E1 (1:1000, rabbit, Cell Signalling #20808): WB manufacturer's website
DENR (WB and IF: 1:2000, guinea pig, in-house production): KO cells within this publication (WB, IF)
pDENR_Ser73 (WB: 1:500, IF: 1:200, rabbit, custom-made by innovagen AB, Lund, Sweden): KO cells within (WB) and besides (IF) this publication
DUSP4 (WB: 1:1000, IF: 1:500, rabbit, abcam ab216576): WB and IF manufacturer's website, IF siRNA-mediated knockdown
FLAG (1:1000, rabbit, Sigma #F7425): WB manufacturer's website
FLAG-M2 (1:1000, mouse, Sigma #F3165): WB manufacturer's website
GAPDH (1:2000, rabbit, Cell Signallling #2118): WB manufacturer's website
gamma-Tubulin (1:1000, Abcam #27074): IF manufacturer's website
Geminin (1:1000, rabbit, Cell Signalling #52508): WB manufacturer's website
pHH3 (Serine 10) (for WB: 1:500, rabbit, Cell Signaling #9701): WB manufacturer's website
pHH3 (Serine 10) (for flow cytometry: 1:100, mouse, Cell Signalling #9706S): flow cytometry manufacturer's website
MAP2K6 (WB: 1:1000, IF: 1:500, rabbit, Cell Signalling #8550): WB manufacturer's website, IF siRNA-mediated knockdown
MCTS1 (1:1000, guinea pig, in-house production): KO cells in our lab
a-raf (1:1000, rabbit, Cell Signaling #4432): WB manufacturer's website
c-raf (1:1000, rabbit, Cell Signaling #9422) : WB manufacturer's website
ERK1/2 (1:1000, rabbit, Cell Signaling #4695) : WB manufacturer's website
GST-HRP conjugate (1:5000, goat, GE Healthcare #RPN1236) : WB manufacturer's website
pERK1/2 (T202/Y204) (1:1000, rabbit, Cell Signaling #4370) : WB manufacturer's website
p-p90RSK (T359/Y363) (1:1000, rabbit, Cell Signaling #9344) : WB manufacturer's website
RSK1/2/3 (1:1000, rabbit, Cell Signaling #14813) : WB manufacturer's website

Eukaryotic cell lines

Policy information about cell lines

Cell line source(s)

HeLa, MCF7 and U2OS: ATCC

Authentication

HeLa, MCF7 and U2OS cells were not further validated.
DENR_KO HeLa cells were authenticated by sequencing of the genomic locus and Western Blot.

Mycoplasma contamination

All cell lines tested negative for mycoplasma.

Commonly misidentified lines
(See ICLAC register)

No commonly misidentified cell lines were used in this study.

Flow Cytometry

Plots

Confirm that:

☒ The axis labels state the marker and fluorochrome used (e.g. CD4-FITC).

☒ The axis scales are clearly visible. Include numbers along axes only for bottom left plot of group (a 'group' is an analysis of identical markers).

☒ All plots are contour plots with outliers or pseudocolor plots.

☒ A numerical value for number of cells or percentage (with statistics) is provided.

Methodology

Sample preparation	Exponentially growing, untreated HeLa cells were trypsinized, spun down, washed twice in PBS, resuspended in Ethanol 70% and then kept at-20°C for 3hrs to several days. Hence, cells were washed twice in PBS, spun down and resuspended in PBS containing Triton X-100 0.25%. After incubation on ice for 15 minutes, cell were washed again in PBS and resuspended in PBS containing BSA 1% and primary antibodies at the indicated dilutions. Cells were incubated o/n at 4°C during gentle shaking, then washed in PBS, resuspended in PBS containing the indicated fluorescent secondary antibodies at a dilution of 1/250 and incubated at room temperature in the dark for 30 minutes during gentle shaking. Finally cells were washed in PBS again and resuspended in PBS for immediate analysis.
Instrument	guava easyCyte HT
Software	guava Soft 3.3
Cell population abundance	Total cells (no doublets, no debris): 10.000-40.000; Mitotic cells (pHH3 positive): 6.000-16.000; Mitotic apoptosis (CC3 and pHH3 positive): 20-300 cells
Gating strategy	1) FSC vs SSC: gate out doublets and debris<br>2) Yellow-G >10^2.4: CC3 positive<br>3) Green-B > 10^3: pHH3 positive

☒ Tick this box to confirm that a figure exemplifying the gating strategy is provided in the Supplementary Information.
